# Zn^2+^–Imidazole Coordination Crosslinks for Elastic Polymeric Binders in High‐Capacity Silicon Electrodes

**DOI:** 10.1002/advs.202004290

**Published:** 2021-03-02

**Authors:** Jaemin Kim, Kiho Park, Yunshik Cho, Hyuksoo Shin, Sungchan Kim, Kookheon Char, Jang Wook Choi

**Affiliations:** ^1^ School of Chemical and Biological Engineering and Institute of Chemical Process Seoul National University 1 Gwanak‐ro, Gwanak‐gu Seoul 08826 Republic of Korea; ^2^ Department of Materials Science and Engineering Seoul National University 1 Gwanak‐ro, Gwanak‐gu Seoul 08826 Republic of Korea

**Keywords:** carboxymethyl cellulose, dynamic crosslinking, elastic binders, in situ crosslinking, metal–ligand coordination, silicon/carbon composite, supramolecular chemistry

## Abstract

Recent research has built a consensus that the binder plays a key role in the performance of high‐capacity silicon anodes in lithium‐ion batteries. These anodes necessitate the use of a binder to maintain the electrode integrity during the immense volume change of silicon during cycling. Here, Zn^2+^–imidazole coordination crosslinks that are formed to carboxymethyl cellulose backbones in situ during electrode fabrication are reported. The recoverable nature of Zn^2+^–imidazole coordination bonds and the flexibility of the poly(ethylene glycol) chains are jointly responsible for the high elasticity of the binder network. The high elasticity tightens interparticle contacts and sustains the electrode integrity, both of which are beneficial for long‐term cyclability. These electrodes, with their commercial levels of areal capacities, exhibit superior cycle life in full‐cells paired with LiNi_0.8_Co_0.15_Al_0.05_O_2_ cathodes. The present study underlines the importance of highly reversible metal ion‐ligand coordination chemistries for binders intended for high capacity alloying‐based electrodes.

## Graphical abstract

Zn^2+^–imidazole coordination bonds, crosslinked with carboxymethyl cellulose in situ during electrode fabrication, are introduced as a polymeric binder for high‐capacity silicon anodes. The reversible metal–ligand bonds impart high elasticity to the binder network for keeping the electrode's integrity. The robust cycling performance under commercial levels of areal capacity highlights the usefulness of high elasticity involving metal–ligand bonds.

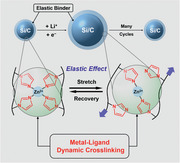

## 1 Introduction

In the battery community, considerable research has led to unambiguous consensus that a polymeric binder plays a critical role in the stable cycling of silicon (Si) anodes in lithium‐ion batteries (LIBs).^[^ ^1^ ^]^ A variety of structural and compositional advancements in active materials, such as glass‐type SiO*_x_* ^[^ ^2^ ^]^ and alloy‐type SiX*_y_* (X = Fe, Mg, etc.)^[^ ^3^ ^]^ based on the adjustment of the elemental composition as well as on smart composite designs^[^ ^4^ ^]^ comprising Si and carbonaceous materials, have enabled the Si content in the electrode to be increased while achieving superior cyclability at the same time. Apart from the advancement in active material design, the incorporation of advanced polymeric binders further improved the key battery performance, thereby raising the status of Si anode technology in its entirety from the standpoint of commercialization.

The promising feature of Si anodes is the possibility they offer to increase the specific energy of a LIB cell by taking advantage of their high theoretical specific capacity (>3000 mAh g^−1^) and low operating potential (≈0.3 V vs Li/Li^+^).^[^ ^5^ ^]^ The increased energy density translates into extending the time for which mobile information technology (IT) devices can be used and the distance electric vehicles (EVs) can be driven per charge. Nevertheless, several failure mechanisms arising from the immense volume change of Si during repeated charge–discharge cycles have long been identified^[^ ^6^ ^]^ as hurdles that need to be overcome. These failure mechanisms include pulverization of particles, delamination of the electrode, and destabilization of the solid‐electrolyte‐interphase (SEI) layer.^[^ ^7^ ^]^ The difficulty with addressing these issues is that these mechanisms are often inter‐related to one another in a way that once one mechanism is triggered, the other mechanisms are increasingly likely to occur.

Besides advanced binder designs,^[^ ^1a^ ^]^ a variety of strategies have been introduced to overcome the aforementioned problems of Si electrodes, targeting active material and electrolyte. From an active material design viewpoint, diverse nanostructures^[^ ^8^ ^]^ and composite designs^[^ ^9^ ^]^ were reported to buffer the volume change of Si. Additive engineering^[^ ^10^ ^]^ is most representative for electrolyte‐based approaches, and in this direction, fluorine‐containing compounds^[^ ^11^ ^]^ are remarkable to produce compact and stable SEI layer.

Clearly, binder design needs to take the structure and size of the active material into consideration. Although diverse nanostructured Si (nano‐Si)^[^ ^12^ ^]^ with different morphologies has been studied widely, its low tap density, high cost, and large surface‐to‐volume ratio render it less attractive for use in practical cells. Instead, micrometer‐sized silicon/carbon (Si/C) composites, and SiO*_x_* have been used most widely for commercial LIBs. Therefore, binder designs targeting these two classes of active material are of utmost priority.

The suitability of a polymeric binder is mainly assessed by its contribution to maintaining the structural integrity of the corresponding electrode. In this context, each polymer can be viewed by focusing on its functionality and chain structure.^[^ ^1a^ ^]^ From the viewpoint of functionality, active particle‐to‐binder interaction with high affinity is preferable to weak van der Waals interaction to sustain the electrode during cycling. From the perspective of the chain structure, 3D network structures are more favorable than their linear counterparts because of the superior ability of 3D networks to dissipate the stress created during the volume expansion of Si.^[^ ^1b^ ^]^ On a related note, the self‐healing capability was recently recognized^[^ ^13^ ^]^ as a useful concept to pursue because it can restore particle‐to‐binder interactions even when these interactions are disrupted when the volume of Si changes. For this purpose, noncovalent bonds with high binding affinity and highly elastic polymer structures were found^[^ ^14^ ^]^ to be effective. It is worthwhile noting that the binder system that is currently the most widely adopted in industry, and thus serves as a reference, is a styrene butadiene rubber/carboxymethyl cellulose (SBR/CMC) hybrid. Its popularity is attributable to the complementarity of its components to each other and their compatibility with the existing aqueous slurry‐based manufacturing scheme. The advantages of this hybrid system are that CMC is highly adhesive to the carbonaceous surface and is suitable for controlling the viscosity of the slurry, while the SBR acts as a cushion to buffer the volume change of the active material. Nonetheless, when the Si content surpasses a certain portion (i.e., 10 wt%), unfortunately, the SBR/CMC binder is no longer able to sustain the integrity of the electrode. This shortcoming calls for the development of more advanced binder designs.

Along this line, various concepts including the implementation of polar functional groups,^[^ ^15^ ^]^ host–guest interactions,^[^ ^16^ ^]^ 3D cross‐linked structures,^[^ ^17^ ^]^ and highly elastic polymer structures^[^ ^18^ ^]^ were recently demonstrated to extend the cycle life markedly. Especially, supramolecular chemistries turned out to be effective in maintaining the electrode integrity because their reversible noncovalent bonds can dynamically restore active particle‐to‐binder and binder‐to‐binder contacts, thus realizing self‐healing. Among the various available noncovalent bonds, our investigation focuses on metal–ligand (M–L) coordination bonds. Our design was motivated by the fact that M–L bonds are one of the strongest noncovalent bonds, and their complexation is well defined based on the specific binding of a central metal ion with a limited pool of designated ligands.^[^ ^19^ ^]^ Thus, once incorporated into an appropriately designed structure, metal ions can be coordinated to carefully selected ligands that protrude from polymer chains and can perform as the centers of dynamic crosslinks in a polymer network.^[^ ^20^ ^]^ We particularly intended to adhere to CMC, as its role as a viscosity enhancer is well established in the industry. Another reason for choosing CMC is to take advantage of its water solubility.^[^ ^21^ ^]^ With respect to the detailed design of the polymer, Zn(II)–imidazole coordination chemistry and its in situ crosslinking capability were exploited and applied to the CMC polymer such that the elasticity/plasticity of the final binder network could be tuned in a sophisticated manner.^[^ ^22^ ^]^ As a result of this binder design, when applied to a micrometer‐sized Si/C composite electrode with a substantial areal capacity (>3 mAh cm^−2^), stable cycling performance was achieved in both half‐cell and full‐cell configurations.

## 2 Results and Discussion

**Figure 1:**
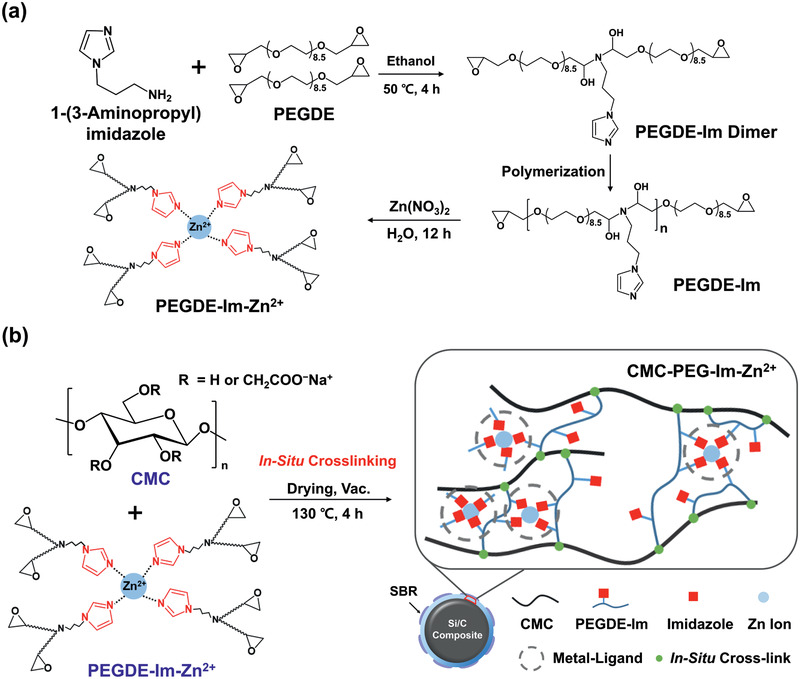
a) Synthetic scheme for the preparation of the metal–ligand complex **PEGDE‐Im‐Zn^2+^**. b) Graphical illustration of the supramolecular network on the Si/C composite formed by in situ crosslinking between CMC and **PEGDE‐Im‐Zn^2+^**.

**Figure** **1**a illustrates the overall synthetic scheme to produce the M–L network. To this end, poly(ethylene glycol) diglycidyl ether (PEGDE) was first reacted with 1‐(3‐aminopropyl) imidazole in a molar ratio of 2:1 via an epoxy‐amine reaction to yield **PEGDE‐Im** dimers.^[^ ^23^ ^]^ The terminal epoxy groups in the dimers were subsequently reacted with 1‐(3‐aminopropyl) imidazole to form **PEGDE‐Im**. Zn^2+^ was then added to yield **PEGDE‐Im‐Zn^2+^** in which Zn^2+^ and imidazole were coordinated in a 1:4 molar ratio. **CMC‐PEG‐Im‐Zn^2+^** was finally obtained through a crosslinking reaction between **PEGDE‐Im‐Zn^2+^** and CMC by exposure to heat. During electrode fabrication, this crosslinking can be achieved when the electrode is being dried (Figure 1b) and thus occurs in situ. The hydroxyl and carboxylate functional groups of CMC can be covalently linked^[^ ^24^ ^]^ with epoxy groups upon heating or under alkaline conditions, as presented in Figure S1 (Supporting Information). Consequently, **CMC‐PEG‐Im‐Zn^2+^** engages in both covalent and noncovalent M–L complex crosslinking, which synergistically maintains the electrode integrity against the massive volume expansion of Si (Figure 1b).

**Figure 2:**
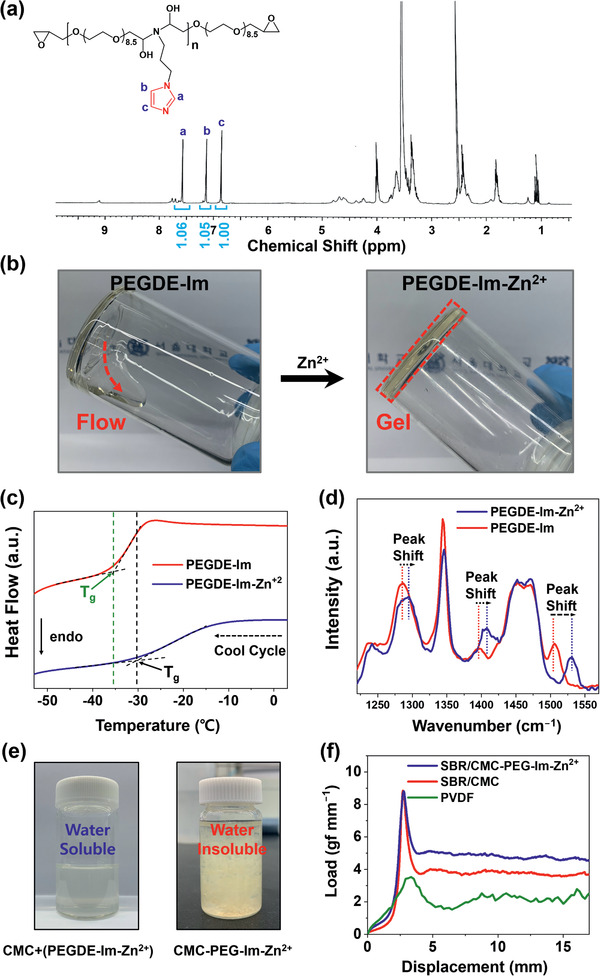
a) ^1^H NMR spectrum of **PEGDE‐Im** (DMSO‐*d*_6_, 400 MHz). b) Digital photographs of (left) **PEGDE‐Im** and (right) **PEGDE‐Im‐Zn^2+^**. c) Differential scanning calorimetry (DSC) profiles and d) Raman spectra of **PEGDE‐Im** and **PEGDE‐Im‐Zn^2+^**. e) Solubility test of CMC+(**PEGDE‐Im‐Zn^2+^**) in water before (left) and after (right) in situ crosslinking. f) Results of the 180° peeling test of the Si/C electrodes with different binders.

The linkage of imidazole to PEGDE was verified by ^1^H nuclear magnetic resonance (NMR) analysis (**Figure** **2**a). When dissolved in DMSO‐*d*_6_ solvent, chemical shifts at 6.86, 7.14, and 7.58 ppm, which are related to the hydrogen on the imidazole ring, were observed.^[^ ^25^ ^]^ The presence of the epoxy groups in **PEGDE‐Im**, which are required for its crosslinking to CMC, was also confirmed by Fourier‐transform infrared (FT‐IR) spectroscopy (Figure S2a, Supporting Information) and ^1^H NMR analysis (Figure S2b, Supporting Information).^[^ ^26^ ^]^ The FT‐IR spectra of **PEGDE‐Im** displayed peaks at 852 and 946 cm^−1^ that are associated with the bending vibration of the epoxy group, implying that the imidazole and epoxy groups were successfully integrated with the designated polymer architectures. The mean molecular weight of **PEGDE‐Im** was 1310.81 Da (Figure S3, Supporting Information), which infers that the number of units (*n* value) in this polymer is between 2 and 3.

Figure 2b visually captures the Zn^2+^–imidazole complexation upon the addition of Zn^2+^ to **PEGDE‐Im**. Before the complexation, **PEGDE‐Im** had the fluency of a viscous liquid when the vial was held upside down (Figure 2b, left), whereas a yellowish gel formed once Zn^2+^ was added (Figure 2b, right). Moreover, M–L complexation affects the mobility of the polymer chains such that the glass transition temperature (*T*_g_) of the polymer is raised. According to the differential scanning calorimetry (DSC) results (Figure 2c), **PEGDE‐Im** and **PEGDE‐Im‐Zn^2+^** exhibited *T*_g_ at −35.9 and −30.2 °C, respectively. The elevated *T*_g_ of **PEGDE‐Im‐Zn^2+^** is attributed to weakened chain mobility owing to crosslinking via M–L complex formation.^[^ ^27^ ^]^ The chemical bonds were further investigated using Raman spectroscopy (Figure 2d; Figure S4a, Supporting Information). M–L complexation in **PEGDE‐Im** manifested itself in the form of peak shifts from 1284, 1398, and 1506 cm^−1^ to 1294, 1408, and 1531 cm^−1^, respectively (Figure 2d). The blue shifts can be explained^[^ ^28^ ^]^ by the strengthened chemical bonds of the imidazole ring, including the C=C bond, as Zn^2+^ coordinates to the imidazole. In fact, it is well accepted that M–L causes the wavenumbers corresponding to the imidazole ring to undergo blue shift by 5–20 cm^−1^ compared with those of its metal‐free configuration.^[^ ^28^, ^29^ ^]^ The peaks of the Zn^2+^–imidazole complex, when not bonded to PEGDE, were consistently observed to undergo blue shift (Figure S4b, Supporting Information). The synthesis of **PEGDE‐Im‐Zn^2+^** was further validated by FT‐IR and ^1^H‐NMR analyses (Figure S5, Supporting Information). In addition, scanning electron microscopy‐energy dispersive spectrometry (SEM‐EDS) (Figure S6, Supporting Information) indicates that Zn is uniformly distributed in **PEGDE‐Im‐Zn^2+^**, and its content was found to be 42.15 g L^–1^ by inductively coupled plasma‐atomic emission spectrometry (ICP‐AES).

As described above, **PEGDE‐Im‐Zn^2+^** is designed to crosslink with CMC in situ upon the application of thermal treatment to dry the electrode. The existence of thermal crosslinking was reflected in the solubility of this compound in water. Interestingly, a blend of **PEGDE‐Im‐Zn^2+^** and CMC was fully water‐soluble (Figure 2e, left), whereas their thermally crosslinked form remained water‐insoluble even after stirring for 3 days (Figure 2e, right). This insolubility can be understood on the basis of the epoxy‐to‐carboxylate and epoxy‐to‐hydroxyl covalent interactions that impede the dispersion of the individual polymer chain in CMC‐PEG‐Im‐Zn^2+^, transforming the entire solution into a gel‐like substance.^[^ ^30^ ^]^ To check the stability of M–L complexation after the crosslinking to CMC, Raman, and FT‐IR analyses were conducted (Figures S7 and S8, Supporting Information). According to the Raman spectroscopy results, a similar level of blue shift characteristic of Zn^2+^‐Im complexation was observed for both before and after the crosslinking to CMC (Figure 2d; Figure S7, Supporting Information), suggesting high stability of M–L complex throughout the synthesis. On the other hand, the FT‐IR spectra of CMC, **CMC‐PEG‐Im**, and **CMC‐PEG‐Im‐Zn^2+^** consistently exhibited characteristic peaks of the carboxylate in CMC at 1590 and 1406 cm^–1^, implying that the carboxylate in CMC remained unchanged during the synthesis.

To verify the stability of the M–L complex during electrode fabrication, the electrode containing **CMC‐PEG‐Im‐Zn^2+^** was examined by X‐ray photoelectron spectroscopy (XPS) and its XPS profiles are presented in Figure S9 (Supporting Information). The **CMC‐PEG‐Im‐Zn^2+^** electrode displayed a peak at 1022.2 eV in its Zn 2p_3/2_ spectra, indicating^[^ ^31^ ^]^ that Zn^2+^ in **CMC‐PEG‐Im‐Zn^2+^** maintained its bivalency during electrode fabrication and supporting the robust character of the M–L complex in our binder. Additionally, inductively coupled plasma‐mass spectrometry (ICP‐MS) showed that the SBR/**CMC‐PEG‐Im‐Zn^2+^** electrode contained a concentration of 300.97 mg kg^–1^ of Zn^2+^. This beneficial property of the M–L complex translated into the mechanical stability of the electrode. Assessment of the adhesion of electrodes via a 180° peeling test over a displacement of 5–15 mm indicated that the adhesion force of the SBR/**CMC‐PEG‐Im‐Zn^2+^** electrode was higher than that of the SBR/CMC‐ and poly(vinylidene fluoride) (PVDF)‐based ones. Specifically, the adhesion force of the SBR/**CMC‐PEG‐Im‐Zn^2+^**, SBR/CMC, and PVDF electrodes was 4.83, 3.79, and 2.07 gf mm^−1^, respectively (Figure 2f). The superior adhesion of the SBR/**CMC‐PEG‐Im‐Zn^2+^** results from the M–L crosslinks that tighten the active particle network, in addition to the well‐known interaction of CMC with the carbon surface of Si/C.^[^ ^1a^ ^]^ The distributions of Si and carbon in the Si/C composite are presented in Figure S10 (Supporting Information).

Considering that the mechanical properties of the binder play a crucial role in maintaining the integrity of the electrode, nanoindentation analysis was carried out for bare polymer films. When subjected to a nanoindentation test, a polymer film generally deforms in one of the three possible ways: plastic, elastic, or viscoelastic (Figure S11, Supporting Information).^[^ ^32^ ^]^ In the case of plastic deformation, the absence of recovery during unloading is represented by a vertical profile on the load versus indentation depth graph. By contrast, elastic deformation involves the opposite behavior such that the loading and unloading profiles overlap, reflecting the perfect reverse trajectory of unloading compared to that of loading. Viscoelastic behavior lies between plastic and elastic deformation, leading to a curved unloading profile that is offset from the loading profile.

**Figure 3:**
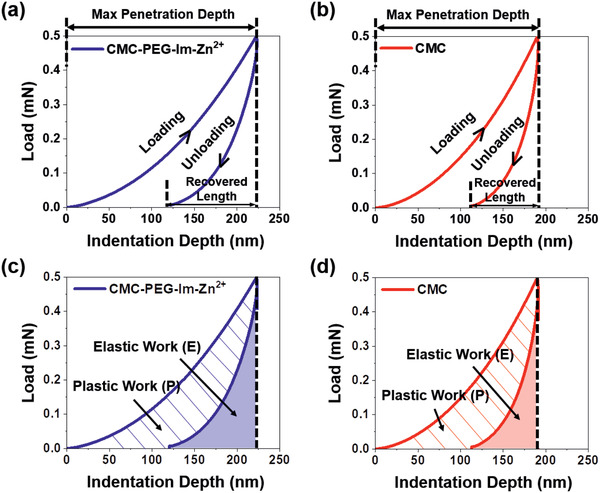
Nanoindentation results of **CMC‐PEG‐Im‐Zn^2+^** (blue) and CMC (red). Load–displacement curves of the a) **CMC‐PEG‐Im‐Zn^2+^** and b) CMC films during a loading–unloading cycle. Maximum penetration depths and recovered lengths are denoted along the *x*‐axes. Load–displacement curves of the c) **CMC‐PEG‐Im‐Zn^2+^** and d) CMC films during the same loading–unloading cycle with plastic work (P) and elastic work (E) noted as areas underneath the curves.

The loading–unloading profiles of **CMC‐PEG‐Im‐Zn^2+^** and CMC with respect to indentation depth are presented in **Figure** **3**a, b, respectively. The maximum penetration depth is referred to as the indentation depth when the maximum load (0.5 mN in our case) is reached, whereas the recovered length is defined as the restored distance upon complete unloading. As a descriptor to quantitatively assess the elasticity of a polymer film, we define the “elastic recovery ratio” as:

(1) $$\mathrm{elastic}\;\mathrm{recovery}\;\mathrm{ratio}\;=\frac{\mathrm{recovered}\;\mathrm{length}}{\max\;\mathrm{penetration}\;\mathrm{depth}}\;$$

The **CMC‐PEG‐Im‐Zn^2+^** film (Figure 3a) exhibited an elastic recovery ratio of 0.46, which is higher than that of the CMC film (0.41) (Figure 3b). When the maximum load was increased to 1.0 mN, the **CMC‐PEG‐Im‐Zn^2+^** film consistently showed a higher recovery: 0.38 versus 0.33 of the CMC film (Figure S12, Supporting Information).^[^ ^33^ ^]^

The elasticity can also be assessed in terms of the degree of energy conservation. As the integrated area below the load versus indentation depth curve corresponds to the energy exerted during the indenting period or the energy restored during the unindenting period, the difference in the integrated areas of the loading and unloading curves (hatched areas in Figure 3c,d) indicate plastic work (P). On the contrary, the integrated areas below the unloading curve represent elastic work (E) (shaded areas in Figure 3c,d). The P and E can also be interpreted as the lost energy absorbed by the material under testing and the energy recovered (thus reversible) by the elasticity of the material, respectively.^[^ ^34^ ^]^ According to this energy analysis, the P and E values of the CMC film were 23.6 and 9.8 pJ, respectively, whereas those of the **CMC‐PEG‐Im‐Zn^2+^** film were 25.6 and 13.9 pJ, respectively. Because the elasticity of a film is related to the relative contributions of these two parameters, the E/P ratio is of interest to us:

(2) $$E/P\;\mathrm{ratio}\;=\frac{\mathrm{elastic}\;\mathrm{work}}{\mathrm{plastic}\;\mathrm{work}}$$

The E/P ratios of the CMC and **CMC‐PEG‐Im‐Zn^2+^** films were 0.41 and 0.54, respectively, for a maximum load of 0.5 mN. A similar tendency was maintained when the load was increased to 1.0 mN (Figure S12, Supporting Information): 0.33 versus 0.41. The well‐known stiff properties of CMC would serve as the origin of the observed lower E/P ratio. By contrast, the Zn^2+^–imidazole complex as well as the poly(ethylene glycol) (PEG) chains endows the polymer network with flexibility, which is responsible for the higher E/P ratio of **CMC‐PEG‐Im‐Zn^2+^**. In particular, the reversible character of the Zn^2+^–imidazole coordination bonds allows for their recovery even when the bonds are perturbed during indentation, which is observed in the form of the large amount of elastic work associated with the corresponding polymer film.

**Figure 4:**
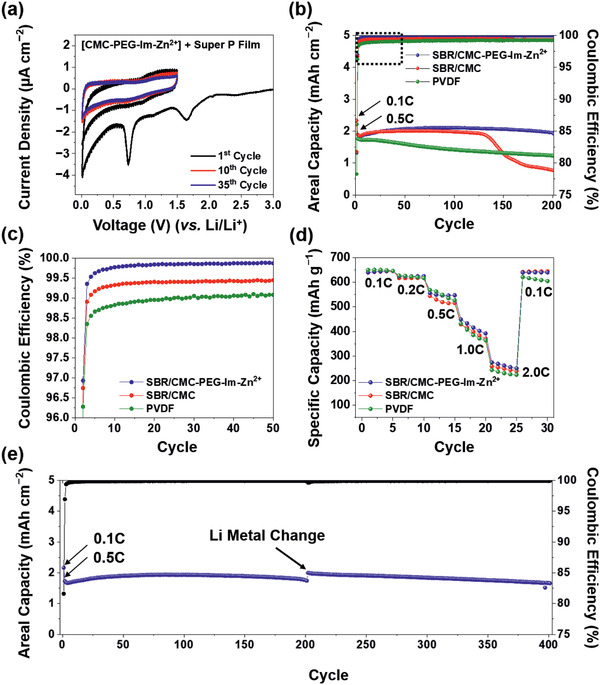
Electrochemical test results of half‐cells using SBR/CMC, SBR/**CMC‐PEG‐Im‐Zn^2+^**, and PVDF binders. a) CV profiles of **CMC‐PEG‐Im‐Zn^2+^** with Super P (1:1 wt%) at 0.05 mV s^−1^ in the potential range of 0.01–1.5 V versus Li/Li^+^. b) Cycling performance of Si/C electrodes using PVDF (green), SBR/CMC (red), and SBR/**CMC‐PEG‐Im‐Zn^2+^** (blue) measured at 0.5 C. c) Corresponding Coulombic efficiencies in the cycle range of 1–50. d) Rate capability at various C‐rates. e) Long‐term cycling performance of the Si/C electrode containing SBR/**CMC‐PEG‐Im‐Zn^2+^** binder when measured at 0.5 C (1.17 mA cm^−2^).

The electrochemical stability of the **CMC‐PEG‐Im‐Zn^2+^** binder was assessed by conducting a cyclic voltammetry (CV) test (**Figure** **4**a). The conductive agent, Super P, was also included in the binder films in a weight ratio of 1:1 to complement the electronic conductivity. The CV profiles of the **CMC‐PEG‐Im‐Zn^2+^** film at the 1st, 10th, and 35th cycle indicate that cathodic peaks at 0.7 and 1.6 V, associated with the decomposition of the electrolyte, are present during the first cycle.^[^ ^35^ ^]^ However, signals related to the reduction of Zn^2+^ were absent, which implies that the Zn^2+^ in the **CMC‐PEG‐Im‐Zn^2+^** remained in the original valence state without undergoing a substantial redox reaction. This phenomenon can also be interpreted to suggest that electron transfer in the Zn^2+^–imidazole complex is limited to the electrode environment. XPS analysis further supports the stable nature of Zn^2+^ during cycling (Figure S13, Supporting Information). Similarly, the robustness of the complex was verified by FT‐IR analysis that showed largely consistent results for the films before and after CV cycles (Figure S14, Supporting Information). Overall, the **CMC‐PEG‐Im‐Zn^2+^** binder proved to be electrochemically inert in the given potential range of the anode (Figure S15, Supporting Information).

To determine the effect of the binder on the key electrochemical performance, Si/C electrodes with different binders were galvanostatically evaluated under the coin half‐cell setting in the potential range of 0.01–1.5 V (vs Li/Li^+^). Each of the electrodes consisted of Si/C active material, binder, and Super P in a weight ratio of 8:1:1. The areal capacity was set to 2.2–2.4 mAh cm^−2^ at 0.1 C (1 C = 650 mA g^−1^) (Figure 4b).^[^ ^36^ ^]^ With this areal capacity, the SBR/**CMC‐PEG‐Im‐Zn^2+^** electrode exhibited sustainable cyclability without any capacity loss at all for 200 cycles when operated at 0.5 C. By contrast, the SBR/CMC electrode lost its capacity abruptly at around the 125th cycle presumably owing to electrolyte depletion.^[^ ^18^, ^37^ ^]^ In the case of the PVDF electrode, the capacity decayed steadily from the beginning, which can be explained by the weak van der Waals force that characterizes the interaction of PVDF with Si/C.^[^ ^17a^ ^]^ The capacity retentions of the SBR/**CMC‐PEG‐Im‐Zn^2+^**, SBR/CMC, and PVDF electrodes after 200 cycles were 100.8%, 40.4%, and 69.9%, respectively. The retention of the SBR/**CMC‐PEG‐Im‐Zn^2+^** electrode in excess of 100% is attributed to an interfacial activation process that would require further investigation to fully understand. A similar trend was observed for the Coulombic efficiency (CE) during cycling. The CE of the SBR/**CMC‐PEG‐Im‐Zn^2+^** electrode rose more drastically such that the CE at the 2nd, 10th, 20th, and 50th cycles was 96.93%, 99.77%, 99.84%, and 99.87%, respectively (Figure 4c). On the contrary, the CEs of the SBR/CMC and PVDF electrodes saturated at 99.46% and 99.09%, respectively, after the 50th and 60th cycles. The average CEs of the SBR/**CMC‐PEG‐Im‐Zn^2+^**, SBR/CMC, and PVDF electrodes for cycles 61–200 were 99.91%, 99.42%, and 99.20%, respectively. The superior CE of the SBR/**CMC‐PEG‐Im‐Zn^2+^** electrode stems from the intimate active particle‐to‐binder interactions that are maintained even during the immense volume change of Si/C by taking advantage of the elasticity of the binder. These intimate contacts stabilize the SEI layer during cycling with controlled electrolyte decomposition such that electrons from the electrode are merely wasted. To elucidate the role of Zn^2+^ in the M–L complexes, the SBR/**CMC‐PEG‐Im** electrode was tested without Zn^2+^‐coordination (Figure S16, Supporting Information). The capacity of this Zn^2+^‐free electrode decayed sharply at around the 130th cycle, thereby reflecting electrolyte depletion owing to uncontrolled interfacial side reactions and reconfirming the importance of Zn^2+^–imidazole complexation. This sharp decay was reflected in a drop in its CE profile; the CE value of the SBR/**CMC‐PEG‐Im** electrode began to drop at the nearly same cycling point around the 130th cycle (Figure S17, Supporting Information).

The rate capability of all the electrodes was assessed by varying the C‐rate from 0.1 C to 2 C (Figure 4d). All the electrodes began operating with similar initial capacities around 640 mAh g^−1^ at 0.1 C, but the SBR/**CMC‐PEG‐Im‐Zn^2+^** electrode was noticeably more effective at preserving the capacity at high C‐rates. For example, at 2 C, the average capacities of the SBR/**CMC‐PEG‐Im‐Zn^2+^**, SBR/CMC, and PVDF electrodes were 261.8, 248.6, and 231.8 mAh g^−1^, respectively. In addition, the capacity of the SBR/**CMC‐PEG‐Im‐Zn^2+^** electrode was fully recovered when the current rate returned to 0.1 C. This superior rate performance is attributed to the elastic nature of the SBR/**CMC‐PEG‐Im‐Zn^2+^** network, which tightens the interparticle contacts to enhance the ionic and electronic transport at the interface.^[^ ^14^, ^18^, ^38^ ^]^ In the same vein, the elasticity of SBR/**CMC‐PEG‐Im‐Zn^2+^** renders the interface of the electrode less prone to degradation during cycling at various C‐rates. Beside the elastic behavior, the PEG units in **CMC‐PEG‐Im‐Zn^2+^** contribute to Li‐ion transport and thus the enhanced rate performance by offering hopping sites for Li ions. This observation was consistent with the electrochemical impedance spectroscopy (EIS) results when measured after 10 cycles (Figure S18, Supporting Information). The SBR/**CMC‐PEG‐Im‐Zn^2+^** electrode produced smaller semicircles corresponding to SEI layer resistance (*R*_SEI_) and charge transfer resistance (*R*_CT_), both of which are critical for good rate performance.

The cycling test was extended to cover a greater number of cycles, namely, 400 (Figure 4e). During this test, the Li metal counter electrode was replaced with a fresh one after 200 cycles to enable us to exclude the effect of the degradation of the Li counter electrode related to Li dendrite growth.^[^ ^18a^ ^]^ The SBR/**CMC‐PEG‐Im‐Zn^2+^** electrode with an areal capacity of 2.16 mAh cm^−2^ retained 96.2% of its initial capacity when cycled at 0.5 C. The average CE in the periods before and after the Li metal replacement was 99.87% and 99.91%, respectively.

**Figure 5:**
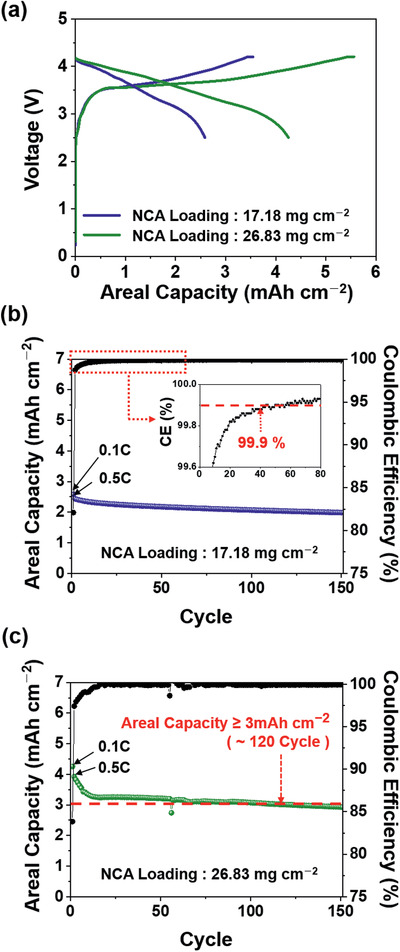
Electrochemical performance of the full‐cell containing the Si/C electrode with SBR/**CMC‐PEG‐Im‐Zn^2+^** binder. a) Initial charge–discharge profiles of Si/C‐LiNi_0.8_Co_0.15_Al_0.05_O_2_ (NCA) full‐cells with different NCA loadings at 0.1 C. Cycling performance of the full‐cells at 0.5 C along with their Coulombic efficiencies over cycling when the NCA loading is b) 17.18 and c) 26.83 mg cm^−2^. The n/p ratios of both cells are 1.1.

The practical viability of SBR/**CMC‐PEG‐Im‐Zn^2+^** was examined by carrying out coin full‐cell tests by pairing the electrode with a LiNi_0.8_Co_0.15_Al_0.05_O_2_ (NCA) cathode with a specific capacity of 183.1 mAh g^−1^ at 0.1 C (Figure S19, Supporting Information). The cathode was composed of NCA, PVDF, and Super P in a weight ratio of 90:5:5, and the active material loading was either 17.18 or 26.83 mg cm^−2^. The full‐cells based on these two different loadings delivered 2.6 and 4.3 mAh cm^−2^ at 0.1 C, respectively (**Figure** **5**a). The capacity ratio between the anode and cathode, namely, the n/p ratio, was set to 1.1. The SBR/**CMC‐PEG‐Im‐Zn^2+^** full‐cell with the lower NCA loading exhibited good cycling behavior such that 81.5% of the original capacity of 2.5 mAh cm^−2^ was retained after 150 cycles at 0.5 C (Figure 5b). This performance was superior to that of the SBR/CMC and SBR/**CMC‐PEG‐Im** full‐cells that preserved 74.6% and 78.2%, respectively, after the same number of cycles (Figure S20, Supporting Information). Remarkably, the CE of the SBR/**CMC‐PEG‐Im‐Zn^2+^** full‐cell rose sharply from the beginning and surpassed 99.9% at the 40th cycle. An SBR/**CMC‐PEG‐Im‐Zn^2+^** full‐cell with the higher NCA loading also preserved 74.7% of the initial capacity after 150 cycles (Figure 5c). It should be noted that the cycle life of full‐cells could be further improved by employing technical know‐how of the various conditions related to cell manufacturing, such as the n/p ratio and viscosity of the slurry. Our study simply aims to demonstrate the practical viability of **CMC‐PEG‐Im‐Zn^2+^**.

**Figure 6:**
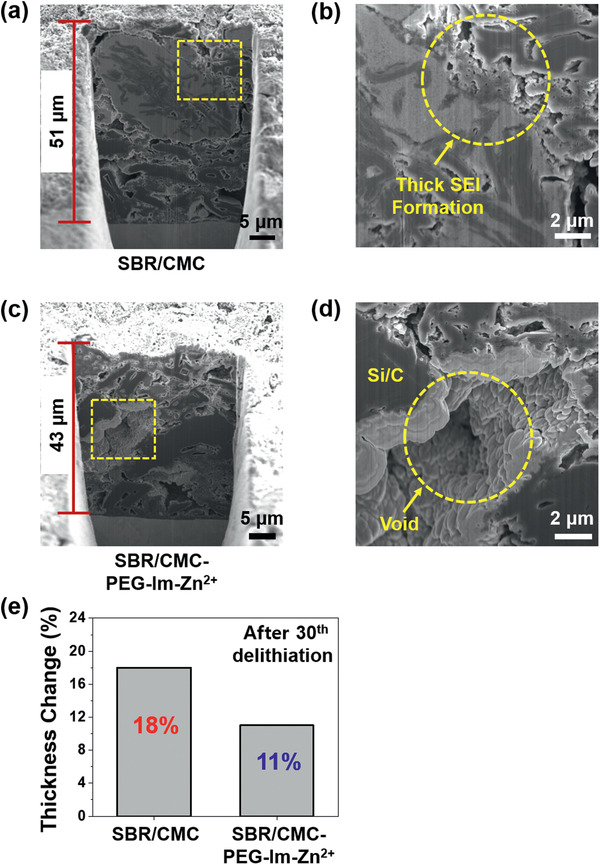
Cross‐sectional SEM images of Si/C electrodes. a) Si/C‐SBR/CMC electrode after the 30th delithiation and b) a magnification of the area within the yellow box. c) Si/C‐SBR/**CMC‐PEG‐Im‐Zn^2+^** electrode after the 30th delithiation and d) a magnification of the area enclosed by the yellow box. e) Thickness changes of the Si/C electrodes after the 30th delithiation.

Cross‐sectional SEM was employed to monitor the thickness and morphology of the SBR/**CMC‐PEG‐Im‐Zn^2+^** electrode during electrochemical cycling (**Figure** **6**). In the pristine state, both the SBR/CMC and SBR/**CMC‐PEG‐Im‐Zn^2+^** electrodes had similar thicknesses of ≈38 µm (Figure S21, Supporting Information). However, the thicknesses of these electrodes became clearly distinct after the 30th delithiation in that they increased to 51 µm (Figure 6a) and 43 µm (Figure 6c), respectively. This change in the relative thickness originated from the different levels of SEI stability,^[^ ^18^, ^39^ ^]^ as revealed by magnified images of the SEI (Figure 6b, d). In the case of the SBR/CMC electrode, the interparticle space was completely filled with the SEI layer (Figure 6b), which provides direct evidence of an unstable interface. By stark contrast, a significant portion of the interparticle space of the SBR/**CMC‐PEG‐Im‐Zn^2+^** electrode remained void (Figure 6d) as the binder contributed greatly to retaining the interparticle contacts. The same trend was observed after 135 cycles (Figure S22, Supporting Information). The bulk‐scale swelling of the electrodes, measured by a micrometer, also reflects the effect of the binder; the thicknesses of the SBR/CMC and SBR/**CMC‐PEG‐Im‐Zn^2+^** electrodes increased by 18% and 11% after 30 cycles, respectively, compared to their pristine states (Figure 6e). It is reminded that electrode swelling is a critical parameter in full‐cell design and thus affects the specific energy of a cell.

**Figure 7:**
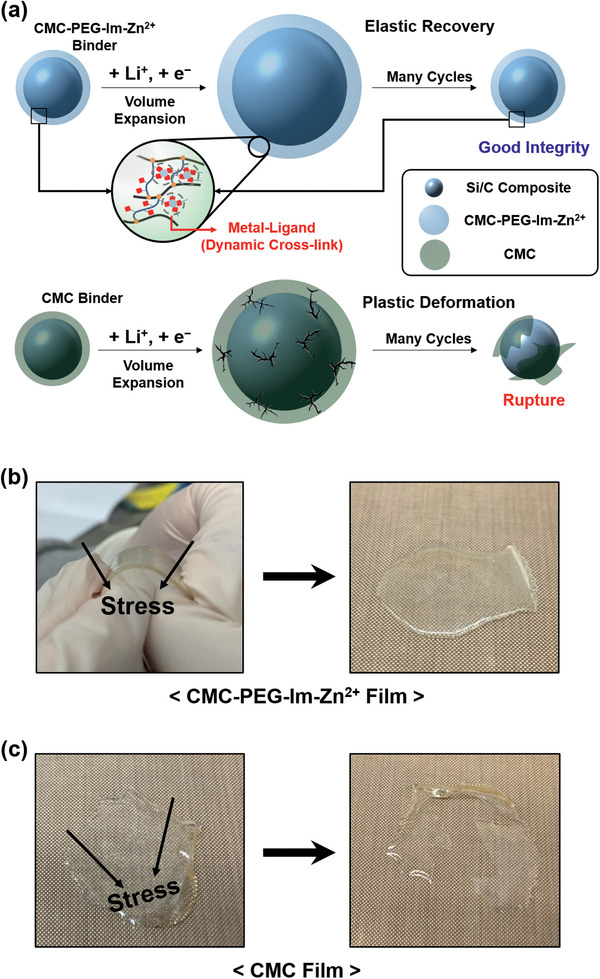
a) Proposed operating mechanisms of the binders in the electrodes during lithiation and delithiation. b,c) Photographs of the **CMC‐PEG‐Im‐Zn^2+^** and CMC films before (left) and after (right) two times of bending–unbending action.

Our combined results serve to corroborate that the superior electrochemical performance of the SBR/**CMC‐PEG‐Im‐Zn^2+^** binder is ascribed to its high elasticity. We portray that this binder network is more effectively able to tolerate the volume change of the active material by utilizing the reversible bonding character of the Zn^2+^–imidazole complex and the flexibility of PEG (**Figure** **7**a). CMC does not have this capability of buffering the volume change of Si because the polymer chains are not chemically interconnected. As a result, upon experiencing repeated charge–discharge cycles, the CMC polymer network ruptures as it is incapable of absorbing the stress originating from the volume change. Hence, “dynamic crosslinking” is the key to the superior performance of the SBR/**CMC‐PEG‐Im‐Zn^2+^** binder. The mechanical robustness of **CMC‐PEG‐Im‐Zn^2+^** was revealed when a film of this compound was subjected to repeated bending–unbending motion. Even after the **CMC‐PEG‐Im‐Zn^2+^** film was repeatedly bent ten times, the original integrity of the film was maintained (Figure 7b; Video S1, Supporting Information). This behavior contrasts that of its CMC counterpart, which ruptured immediately after far weaker elongation stress was applied (Figure 7c; Video S2, Supporting Information).

## 3 Conclusion

In summary, we demonstrated the Zn^2+^–imidazole complex as a useful crosslinking component to realize a binder with outstanding elasticity for Si/C composite electrodes. Crosslinking is accomplished in situ during the drying step of electrode preparation such that the dispersion of the binder throughout the electrode does not present a problem. The high elasticity of **CMC‐PEG‐Im‐Zn^2+^** maintains tight interparticle contacts during cycling and enables the interface to remain stable and to offer high and consistent ionic conductivity. The recoverable nature of Zn^2+^–imidazole coordination bonds is also beneficial for sustaining the electrode integrity during the huge volume change Si/C undergoes, and this benefit is closely linked to its extended cycle life. This study highlights the significance of reversible noncovalent crosslinks as generally useful tools for designing a binder that targets high capacity battery electrodes that are adversely affected by a large volume change.

## 4 Experimental Section

##### Materials and Chemicals

PEGDE (M_n_ = 500), 1‐(3‐aminopropyl) imidazole, Na‐CMC (M_w_ = 250 000), and PVDF (M_w_ = 534 000) were purchased from Sigma‐Aldrich (USA). Ethanol was purchased from Samchun (South Korea). Zinc nitrate hexahydrate (Zn(NO_3_)_2_∙6H_2_O) was purchased from Alfa Aesar (USA). The average particle size of the Si/C composite was 10 µm. Super P was purchased from Timcal (Switzerland). A dialysis membrane tube with a molecular weight cut‐off of 1 kDa was purchased from Membrane Filtration Products, Inc. (USA). *N*‐methyl‐2‐pyrrolidone (NMP) was purchased from Junsei (Japan).

##### Synthesis of Polymer

**PEGDE‐Im** was synthesized by introducing 9 g of PEGDE and 3.6 g of 1‐(3‐aminopropyl) imidazole to 60 mL ethanol and stirred at 50 °C for 4 h. The mixture was dialyzed against ethanol for 3 days using a dialysis membrane (MWCO, 1 kDa) to remove unreacted molecules. After the dialysis, the mixture was lyophilized to yield the final product. **PEGDE‐Im‐Zn^2+^** was synthesized by adding 2.14 g of zinc nitrate hexahydrate to the above‐mentioned solution of **PEGDE‐Im** and stirred for 12 h. The molar ratio of imidazole and Zn^2+^ was 4:1. The same purification method as for **PEGDE‐Im** was used for the mixture and yielded a yellow transparent elastomeric material.

##### Characterization

Field‐emission scanning electron microscopy (FE‐SEM) (JSM‐7800F Prime, JEOL, Japan) was used to characterize the morphology of the various electrodes. Energy dispersive spectrometry (EDS) mapping was performed to visualize the elemental distributions of the active material. ^1^H NMR analysis (Bruker Avance III NMR 400 MHz) and FT‐IR spectroscopy (TENSOR27, Bruker, Germany) were carried out to characterize the chemical structures. The molecular weight distribution and mean value of **PEGDE‐Im** were measured using matrix‐assisted laser desorption ionization mass spectrometer (MALDI‐TOF) (MALDI‐TOF Voyager DE‐STR, Applied Biosystems, USA). The *T*_g_ was determined using DSC (PerkinElmer DSC 4000) at a ramping rate of 10 °C min^−1^. Raman spectra were recorded on a Raman spectrometer II (DXR 2xi, Thermo, USA). For this analysis, polymer films were prepared on a glass slide and a 532 nm laser was used as the light source. ICP‐AES (OPTIMA 8300, Perkin‐Elmer, USA), ICP‐MS (NexION 350D, Perkin‐Elmer, USA), and SEM‐EDS analyses were conducted to evaluate the content of Zn and its distribution in the polymer. The peeling tests were performed using a universal testing machine (QM100s, QMESYS, South Korea). In the peeling tests, 3M double‐sided tape was attached to the electrodes and peeled off at a rate of 25 mm min^−1^ to evaluate the adhesion strength. The surface components of electrodes were analyzed using an X‐ray photoelectron spectrometer (Axis Supra, Kratos, UK). Nanoindentation tests were carried out using an ultra‐precision surface mechanical analyzer (Anton Paar, Austria). For this analysis, samples were prepared by spin‐coating polymers on a Si wafer at 3000 rpm for 100 s, followed by a drying step at 60 °C for 24 h. In the case of **CMC‐PEG‐Im‐Zn^2+^**, the drying procedure differed and entailed drying at 130 °C in a vacuum oven for 4 h and subsequently at 60 °C under vacuum overnight. The indentation load was either 0.5 or 1 mN and the tests were repeated at five different positions on each electrode. Cross‐sectional images of the electrodes were attained using a focused ion beam (FIB) (Helios G4, Thermo Fisher Scientific, USA). Electrodes were first dissected using a gallium (Ga) ion beam at 30 kV, and images of their cross‐sections were captured via FE‐SEM in the same FIB instrument.

##### Preparation of Electrodes

Electrode preparation involved first preparing slurries containing Si/C, binder, and Super P in a weight ratio of 8:1:1. In this process, each binder was first dispersed in deionized water. For the SBR/CMC binder, the weight ratio between SBR and CMC was 1:1. For the SBR/**CMC‐PEG‐Im‐Zn^2+^** binder, the weight ratio among SBR, CMC, and **PEGDE‐Im‐Zn^2+^** was 5:4.5:0.5. The viscosity of the SBR/**CMC‐PEG‐Im‐Zn^2+^** and SBR/CMC slurries with respect to shear rate is presented in Figure S23 (Supporting Information). The slurry for each electrode was cast on copper foil using the doctor blading method. In the case of the SBR/**CMC‐PEG‐Im‐Zn^2+^** and SBR/**CMC‐PEG‐Im** electrodes with active loadings of 3.67 and 3.65 mg cm^−2^, respectively, the electrodes were dried in a vacuum oven at 130 °C for 4 h to allow thermal crosslinking to take place, followed by additional drying at 60 °C under vacuum overnight. The control electrodes based on PVDF and SBR/CMC underwent drying at 100 °C for 10 min and then at 60 °C under vacuum overnight. The active loadings of these control electrodes were 3.62 and 3.67 mg cm^−2^, respectively. All electrodes were compressed to reach the density of 1.0–1.1 g cm^−3^. The assembly of full‐cells required the fabrication of cathodes consisting of NCA, PVDF, and Super P in a weight ratio of 90:5:5. For this electrode fabrication, the slurry based on NMP was cast onto aluminum foil, followed by drying at 60 °C under vacuum for one day. For full‐cell assembly, the N/P ratio was set to 1.1 to provide a proper amount of excessive Li accommodation sites in the anode during the precycle.

##### Electrochemical Measurements

CR2032 coin cells were assembled in an Ar‐filled glove box for all galvanostatic electrochemical tests. The electrode diameter was 10 mm in all cases. In the half‐cells, Li metal foil with a 15 mm diameter was used as the reference/counter electrode. Polyethylene film (SK Innovation, South Korea) was used as a separator. The electrolyte comprised 1.0 m lithium hexafluorophosphate (LiPF_6_) in ethylene carbonate (EC)/diethylene carbonate (DEC) in 1/1 volume ratio with 10 wt% fluoroethylene carbonate (Welcos, South Korea). All electrochemical data were recorded on a battery cycler (WBCS 3000, WonATech, South Korea) at 25 °C. Before cycling, all cells were rested for 6 h. Prior to the half‐cell tests, one precycle was implemented at 0.1 C to form a stable SEI layer, and subsequent cycles were at 0.5 C. The potential range in both the precycle and subsequent cycles was 0.01–1.5 V (vs Li/Li^+^), and each charge and discharge cycle was carried out in constant current (CC) mode. In the rate capability tests, each cell was subjected to various C‐rates from 0.1 to 2.0 C. The full‐cell tests were conducted by using one precycle at 0.1 C and subsequent cycles were scanned at 0.5 C in the potential range of 2.5–4.2 V in constant current constant voltage mode for charging and in CC mode for discharging. EIS was employed using a potentiostat (VSP, Bio‐Logic, France). The frequency ranged from 1 MHz to 0.1 Hz. CV tests were carried out at a scan rate of 0.05 mV s^−1^ in the potential range of 0.01–1.5 V (vs Li/Li^+^).

## Conflict of Interest

The authors declare no conflict of interest.

## Supporting information

Supporting Information

Supplemental Video 1

Supplemental Video 2

## Data Availability

The data that support the findings of this study are available from the corresponding author upon reasonable request.
