# Supplementary material for: Zn2+–Imidazole Coordination Crosslinks for Elastic Polymeric Binders in High‐Capacity Silicon Electrodes
Source: Adv Sci (Weinh). 2021 Mar 2;8(9):2004290. doi: 10.1002/advs.202004290 (PMC8097348; doi:10.1002/advs.202004290)
Supplement: Supplementary file 1 — Supporting Information [file ADVS-8-2004290-s002.pdf]

WILEY-VCH

© 2021 Wiley-VCH GmbH

ADVANCED SCIENCE
Open Access

Supporting Information

for *Adv. Sci.,* DOI: 10.1002/advs.202004290

Zn2+-imidazole Coordination Crosslinks for Elastic Polymeric Binders in High Capacity Silicon Electrodes

*Jaemin Kim, Kiho Park, Yunshik Cho, Hyuksoo Shin, Sungchan Kim, Kookheon Char, and Jang Wook Choi**

© 2021 Wiley-VCH GmbH

Supporting Information

Zn^{2+}-imidazole Coordination Crosslinks for Elastic Polymeric Binders in High Capacity Silicon Electrodes

*Jaemin Kim, Kiho Park, Yunshik Cho, Hyuksoo Shin, Sungchan Kim, Kookheon Char, and Jang Wook Choi**

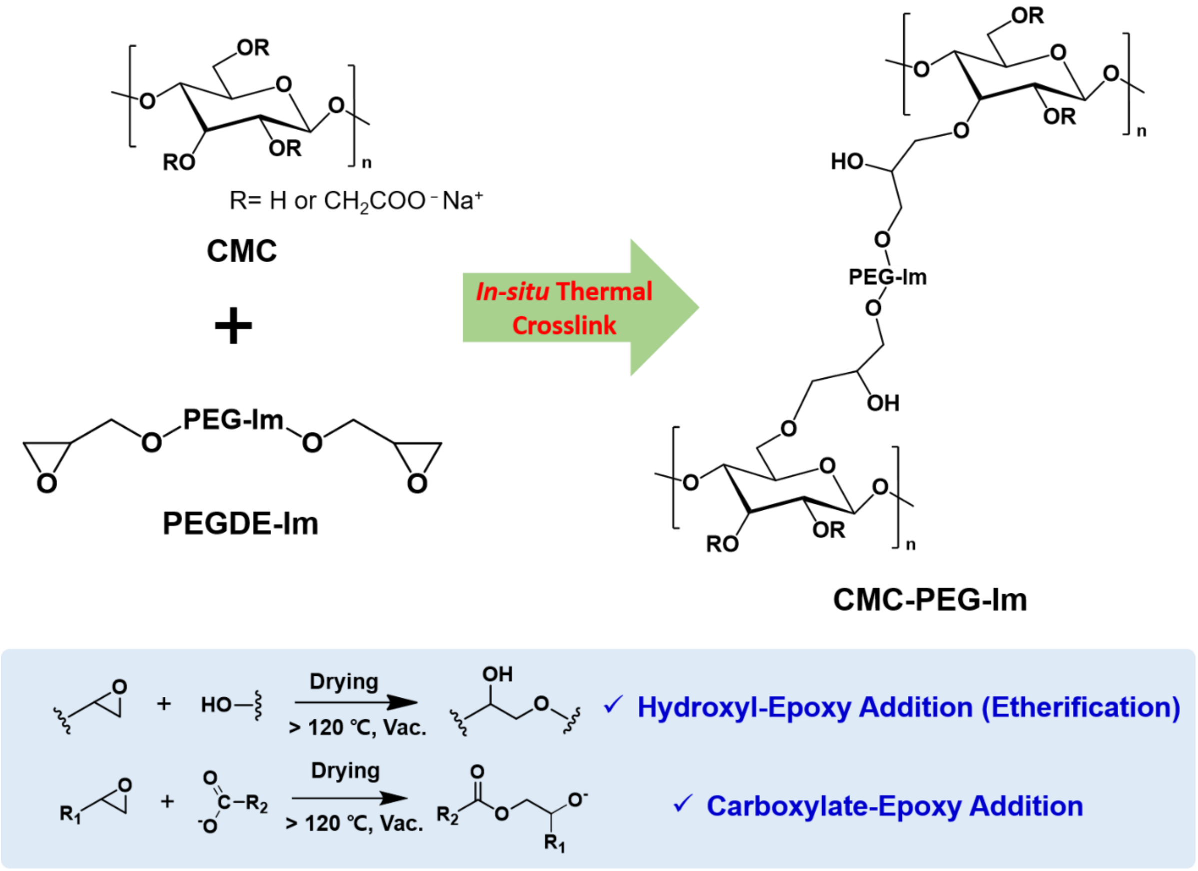

Figure S1. Scheme of the chemical reaction between the hydroxyl and carboxylate groups of CMC and the epoxy group of **PEGDE-Im**.

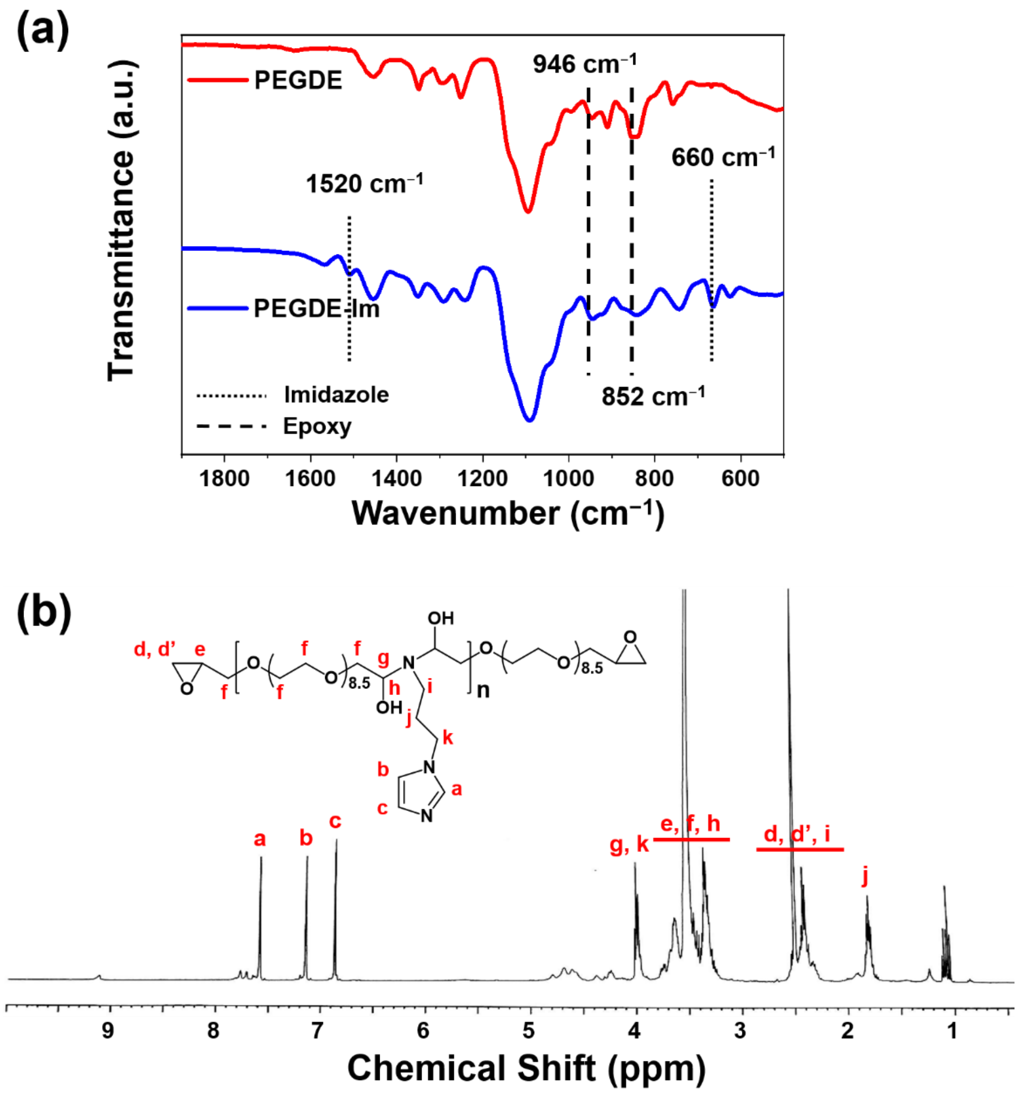

Figure S2. (a) FT-IR profiles of PEGDE and **PEGDE-Im**. (b) ^{1}H NMR spectrum of **PEGDE-Im**.

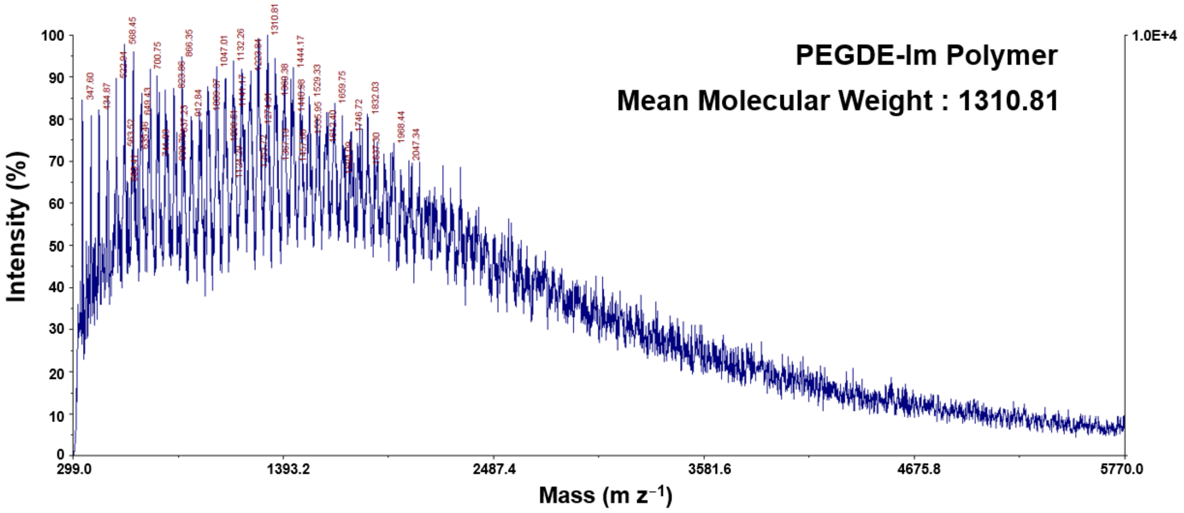

Figure S3. Molecular weight distribution and mean value of **PEGDE-Im**.

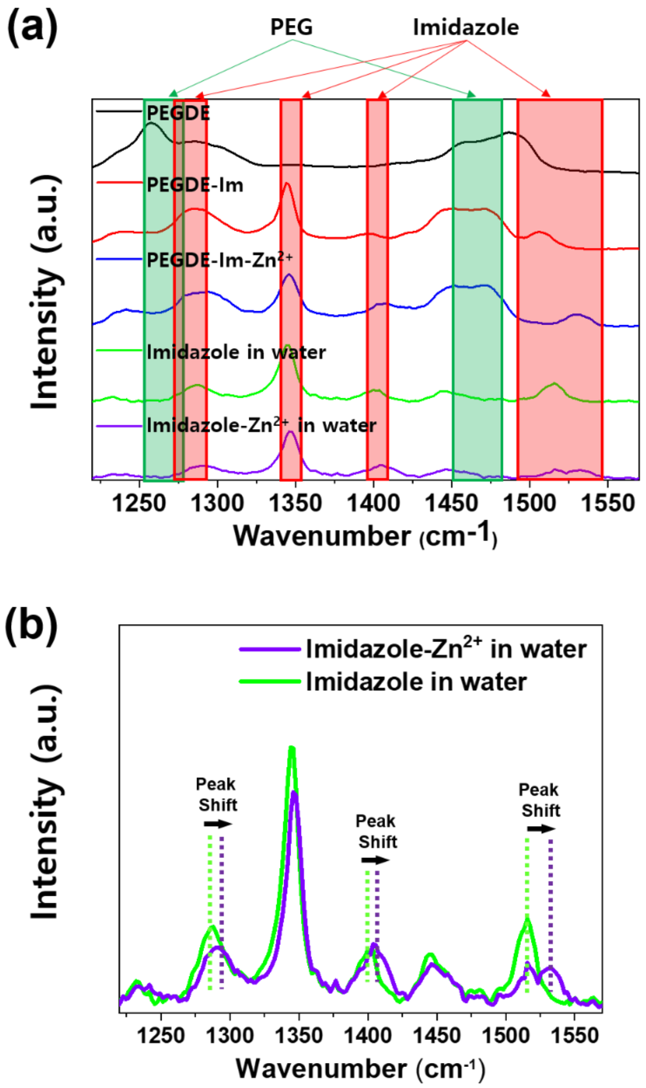

Figure S4. Raman spectra showing (a) PEG and imidazole in **PEGDE-Im** and **PEGDE-Im-Zn^{2+}** and (b) imidazole and imidazole-Zn^{2+} in water.

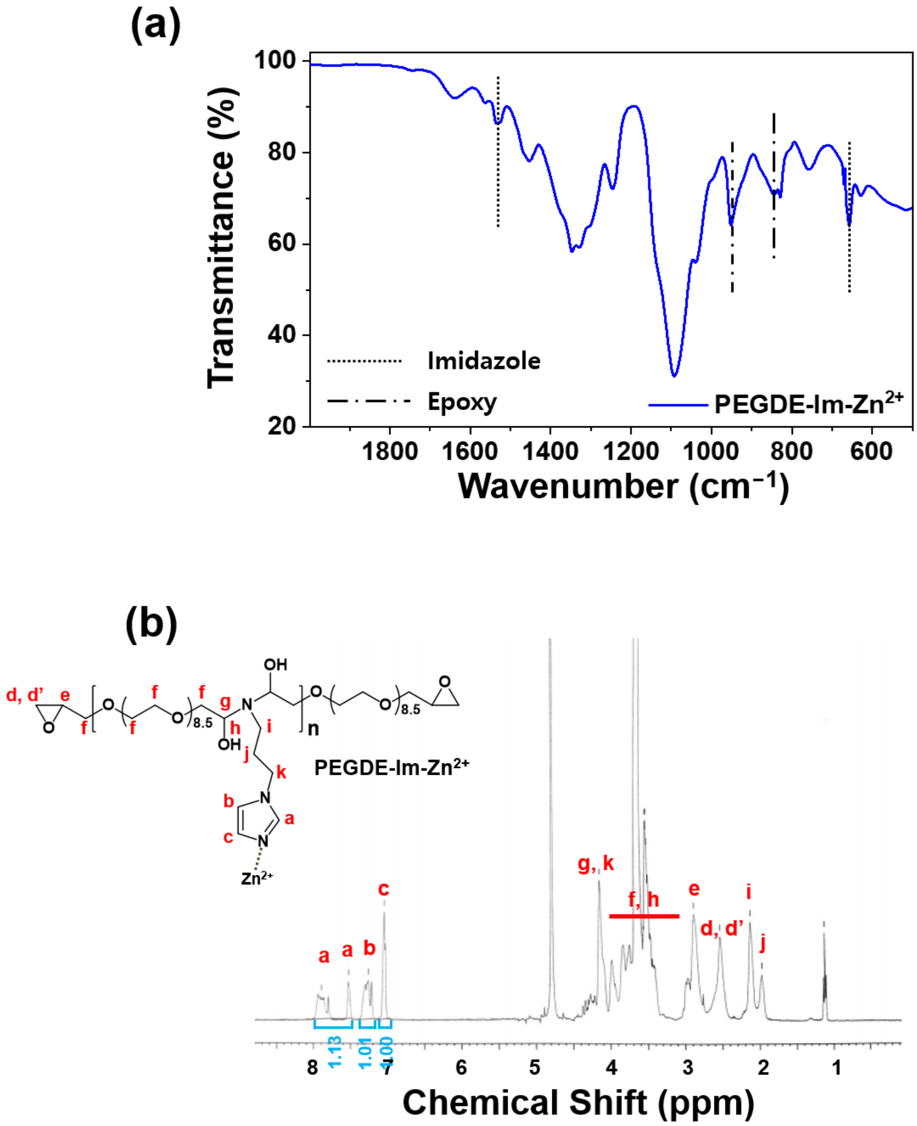

Figure S5. (a) FT-IR and (b) ^{1}H NMR results of **PEGDE-Im-Zn^{2+}**.

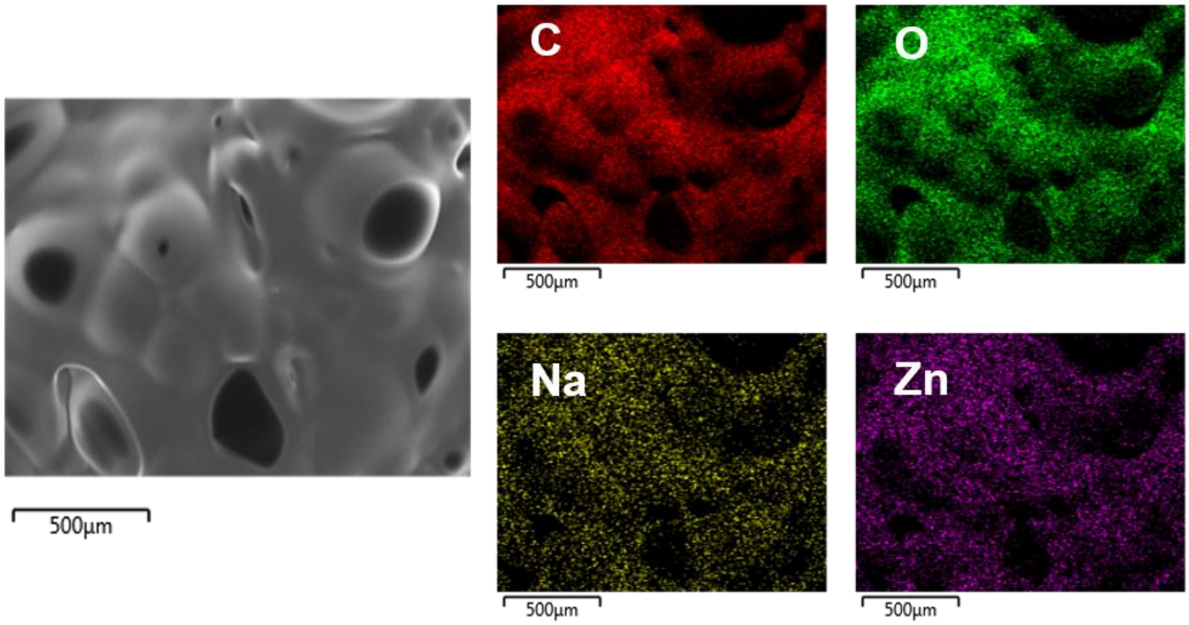

Figure S6. SEM image and corresponding elemental mapping of **PEGDE-Im-Zn^{2+}** using EDS.

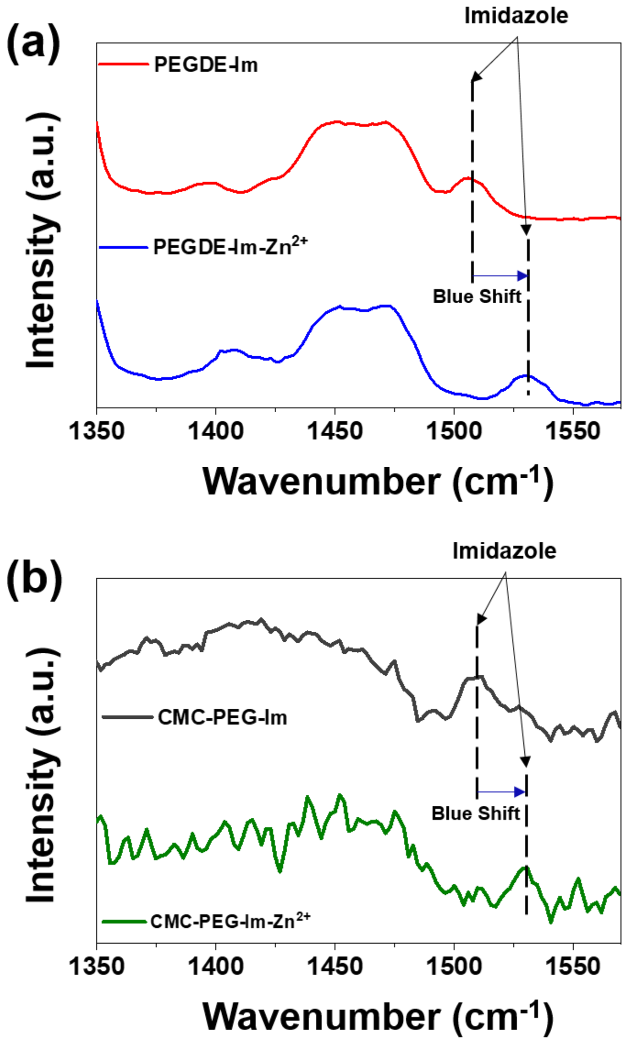

Figure S7. Raman spectra of (a) **PEGDE-Im** and **PEGDE-Im-Zn^{2+}** and (b) **CMC-PEG-Im** and **CMC-PEG-Im-Zn^{2+}**.

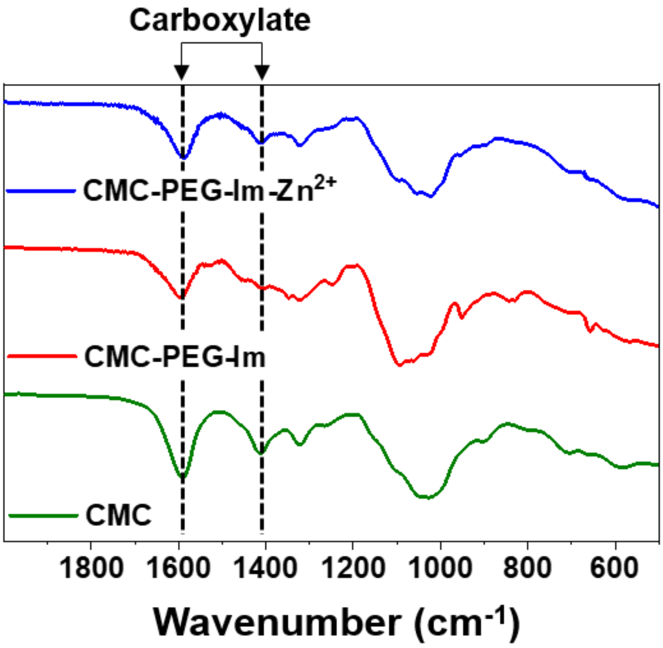

Figure S8. FT-IR spectra of CMC, **CMC-PEG-Im**, and **CMC-PEG-Im-Zn^{2+}**.

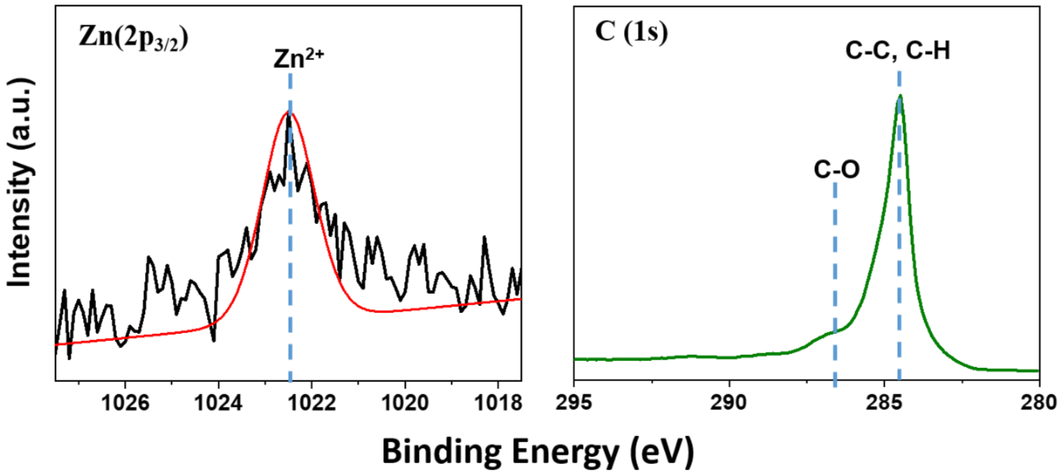

Figure S9. XPS results of the SBR/**CMC-PEG-Im-Zn^{2+}** electrode.

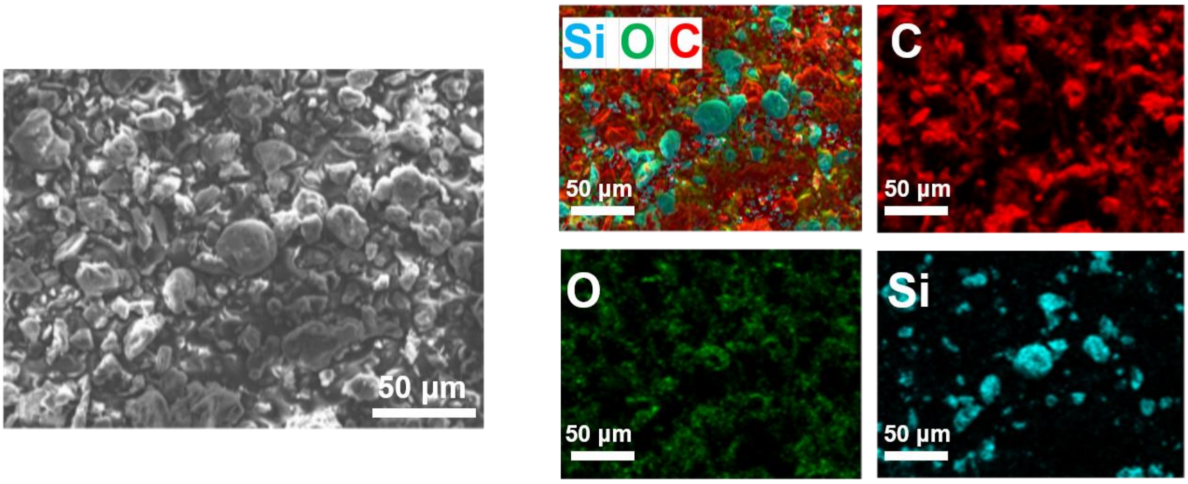

Figure S10. SEM images and EDS elemental mapping of the Si/C composite.

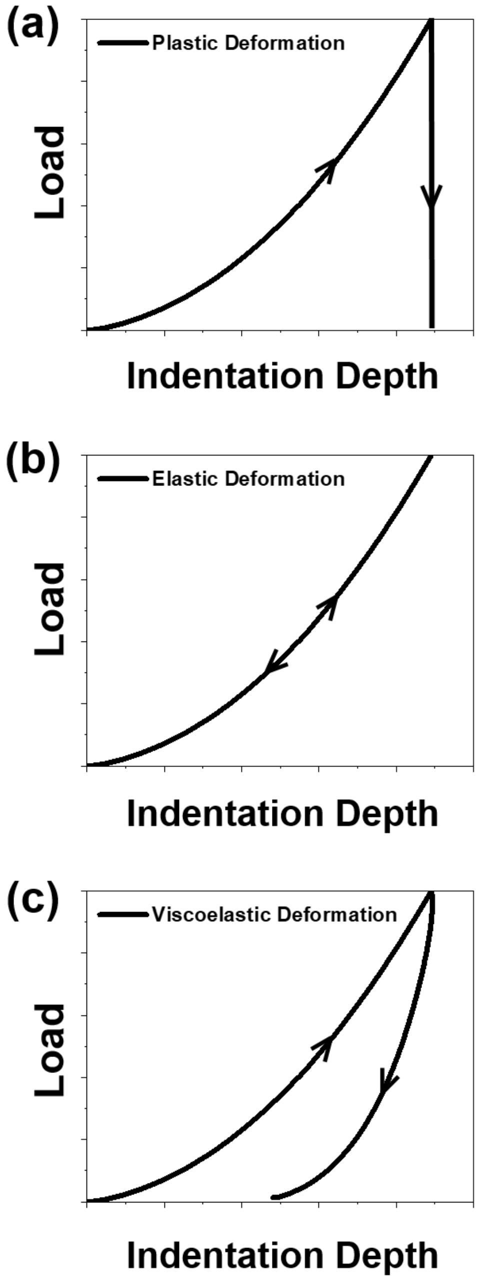

Figure S11. Three possible different types of behavior of films when subjected to nanoindentation. (a) Plastic deformation, (b) elastic deformation, and (c) viscoelastic deformation.

(a)

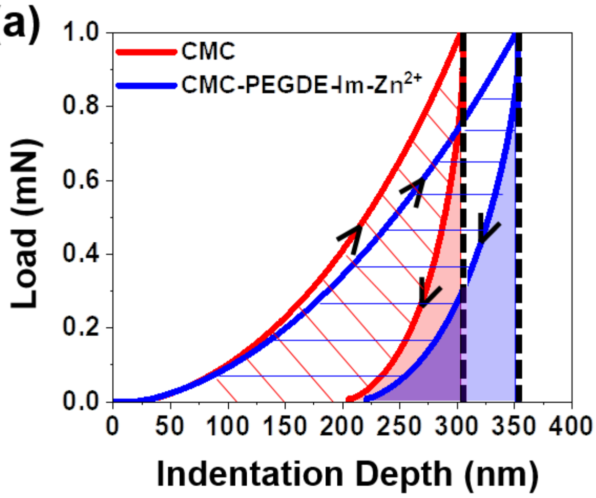

(b)

	Elastic Recovery Ratio	E/P Ratio
CMC	0.33	0.33
CMC-PEG-Im-Zn^{2+}	0.38	0.41

Figure S12. Nanoindentation results of polymer films when the maximum load was set to 1 mN. (a) Load-displacement curves of the CMC and **CMC-PEG-Im-Zn^{2+}** films during one loading-unloading cycle. (b) Elastic recovery ratio and E/P work ratio of the CMC and **CMC-PEG-Im-Zn^{2+}** films.

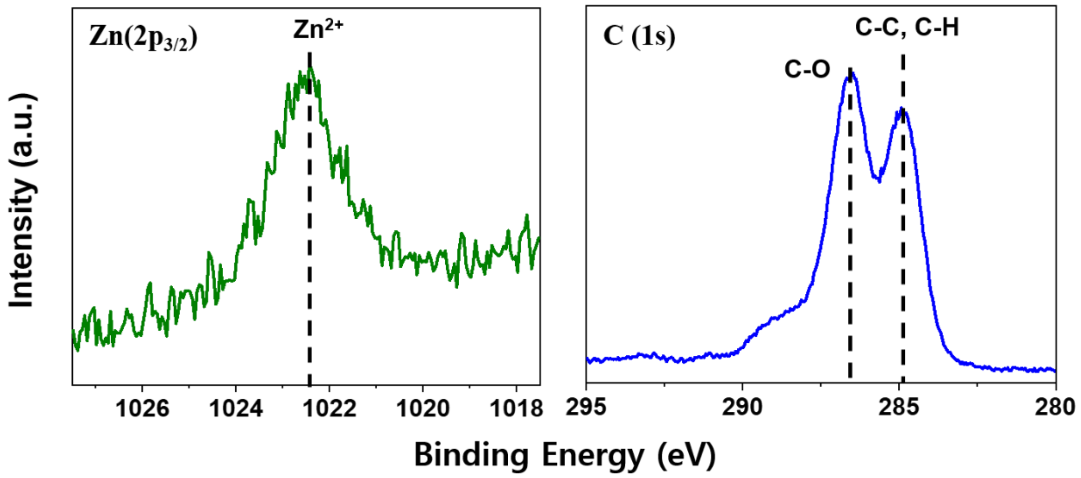

Figure S13. XPS results of the **CMC-PEG-Im-Zn^{2+}** film after 30 CV cycles at 0.5 mV s^{-1} in the voltage range of 0.01−1.5 V.

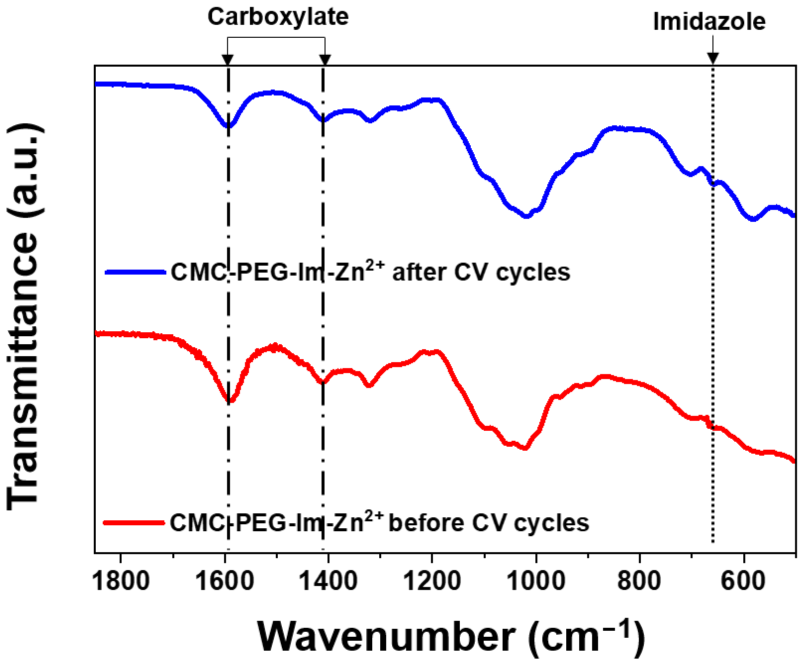

Figure S14. FT-IR spectra of the **CMC-PEG-Im-Zn^{2+}** film before and after 30 CV cycles at 0.5 mV s^{-1} in the voltage range of 0.01−1.5 V.

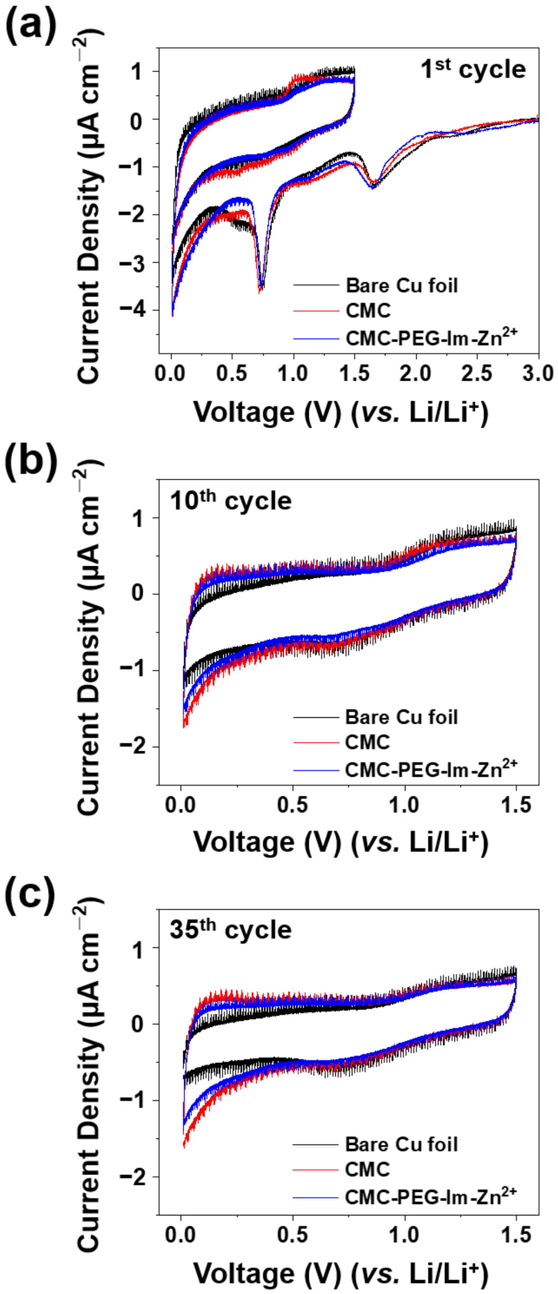

Figure S15. CV profiles of CMC, **CMC-PEG-Im-Zn^{2+}** with Super P (1:1 wt%), and bare Cu foil at the (a) 1st cycle, (b) 10th cycle, and (c) 35th cycle.

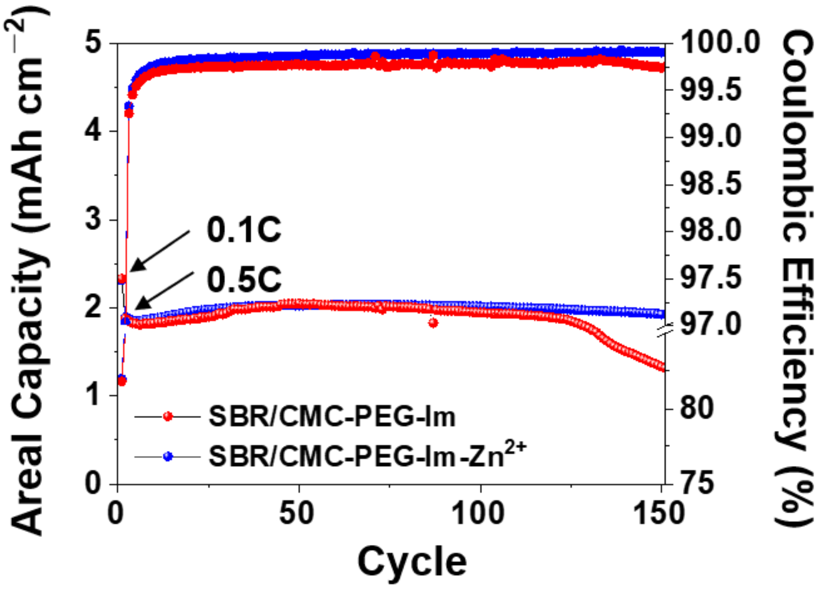

Figure S16. Cycling performance of the Si/C electrodes containing SBR/**CMC-PEG-Im** and SBR/**CMC-PEG-Im-Zn^{2+}**.

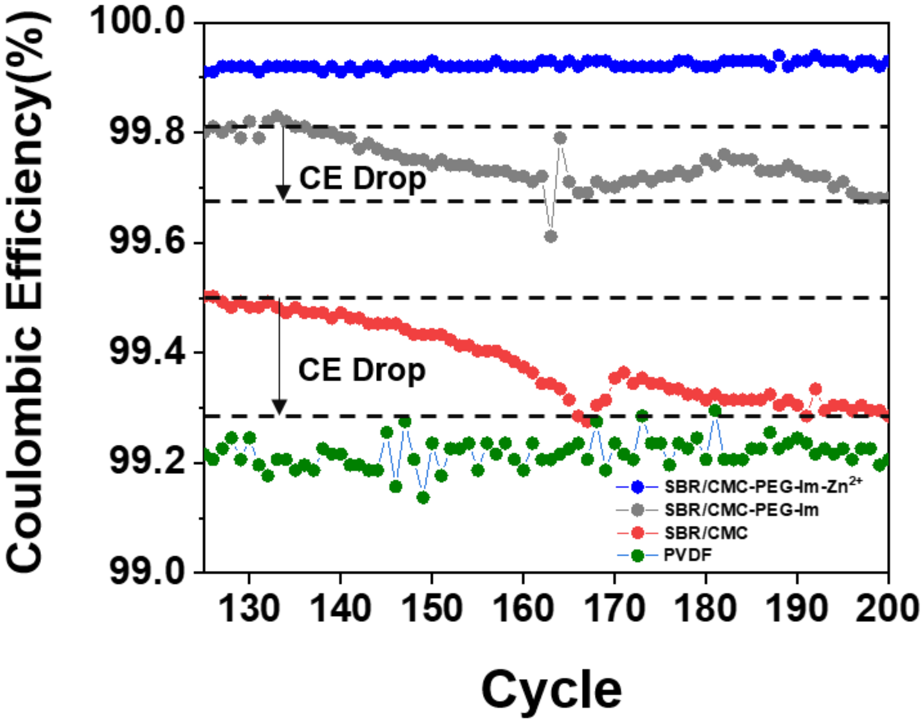

Figure S17. Coulombic efficiencies of the Si/C electrodes containing PVDF (green), SBR/CMC (red), SBR/**CMC-PEG-Im** (gray), and SBR/**CMC-PEG-Im-Zn^{2+}** (blue) in the cycle range of 125−200.

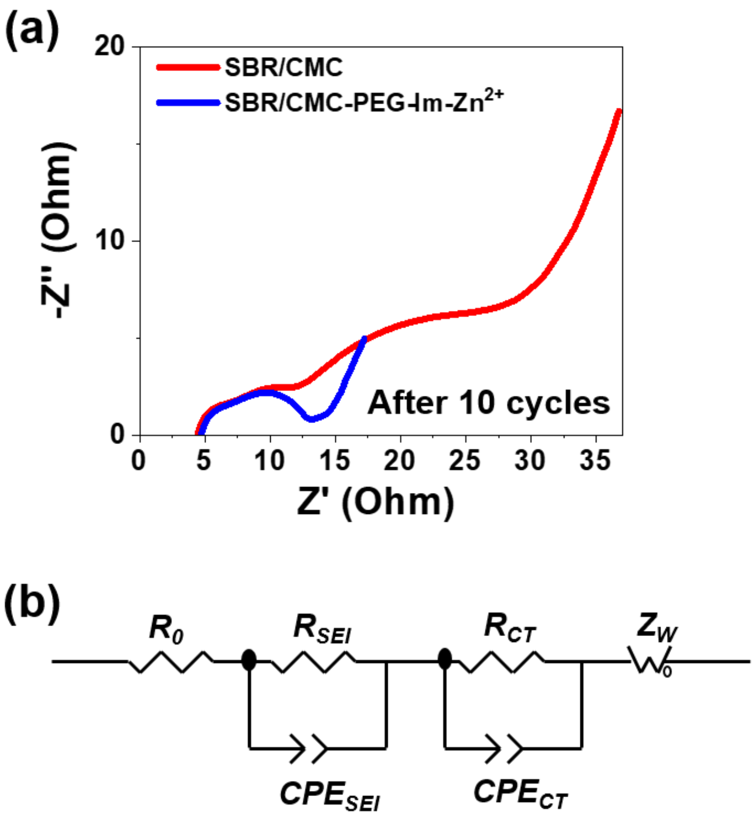

	After 10 Cycles (Ω)		
	R_0	R_{SEI}	R_{CT}
SBR/CMC	4.49	14.49	39.33
SBR/CMC-PEG-Im-Zn^{2+}	4.77	8.92	12.92

Figure S18. (a) EIS fitting of electrodes after 10 cycles at 0.5C. (b) Equivalent circuit for the EIS and corresponding resistance results obtained by fitting to the circuit.

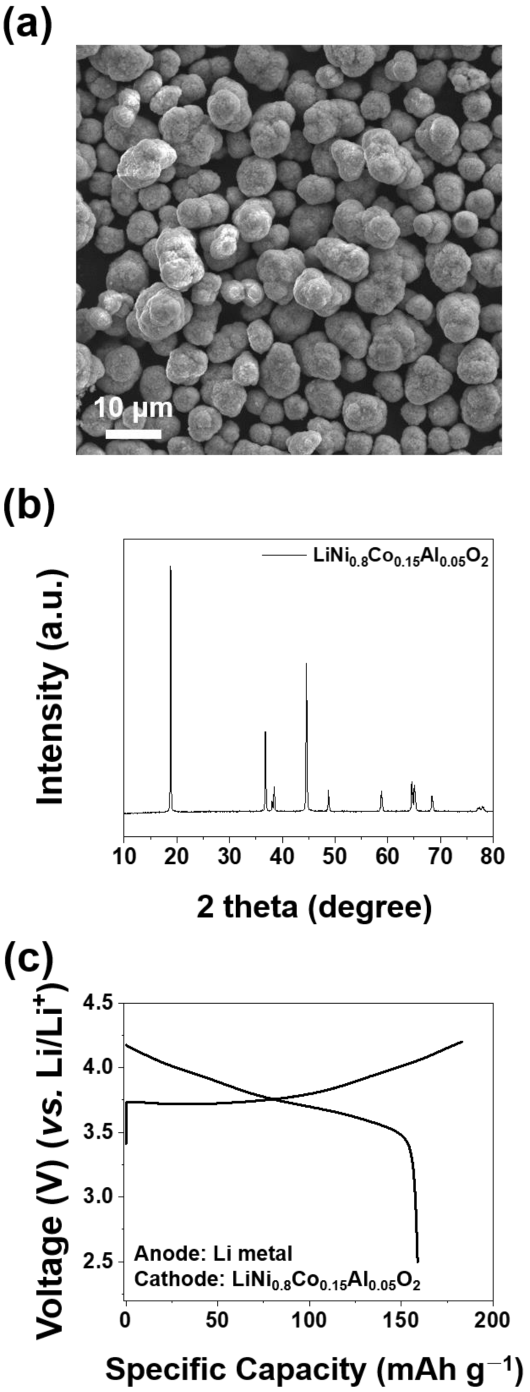

Figure S19. (a) SEM image and (b) XRD pattern of NCA powder. (c) 1st charge-discharge profile of NCA under the half-cell configuration when scanned at 0.1C.

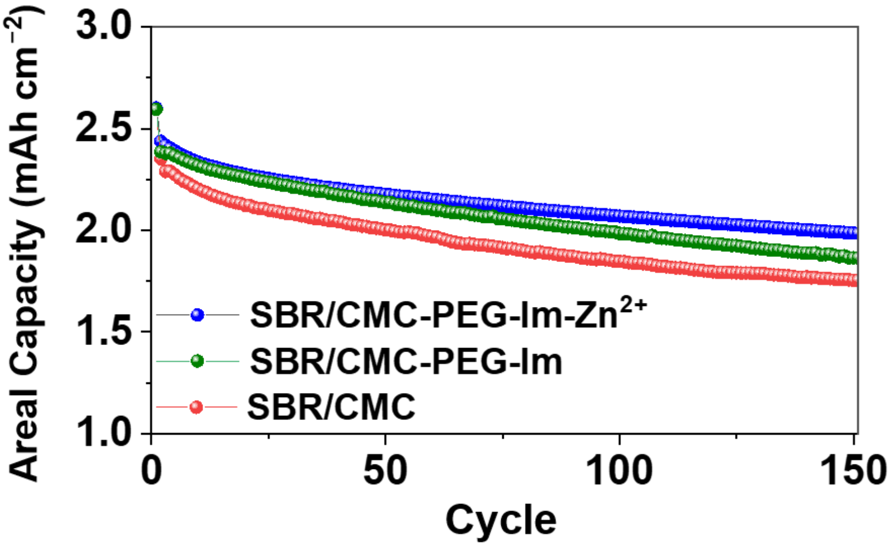

Figure S20. Cycling performance of the full-cells based on the SBR/**CMC-PEG-Im-Zn^{2+},** SBR/**CMC-PEG-Im,** and SBR/CMC binders.

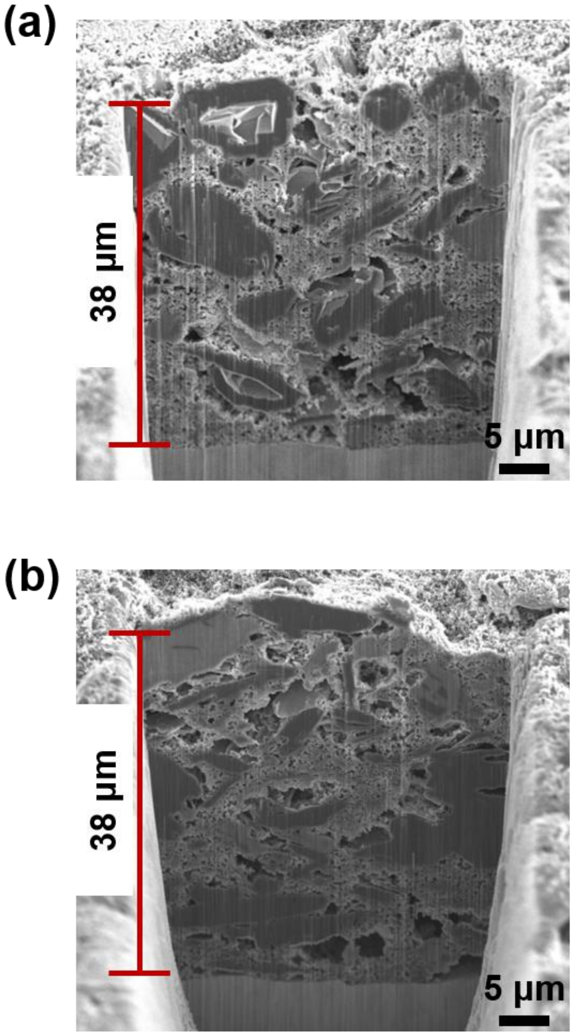

Figure S21. Cross-sectional SEM images of the (a) Si/C-SBR/CMC and (b) Si/C-SBR/**CMC-PEG-Im-Zn^{2+}** electrodes in the pristine state. The thicknesses of both electrodes were 38 µm.

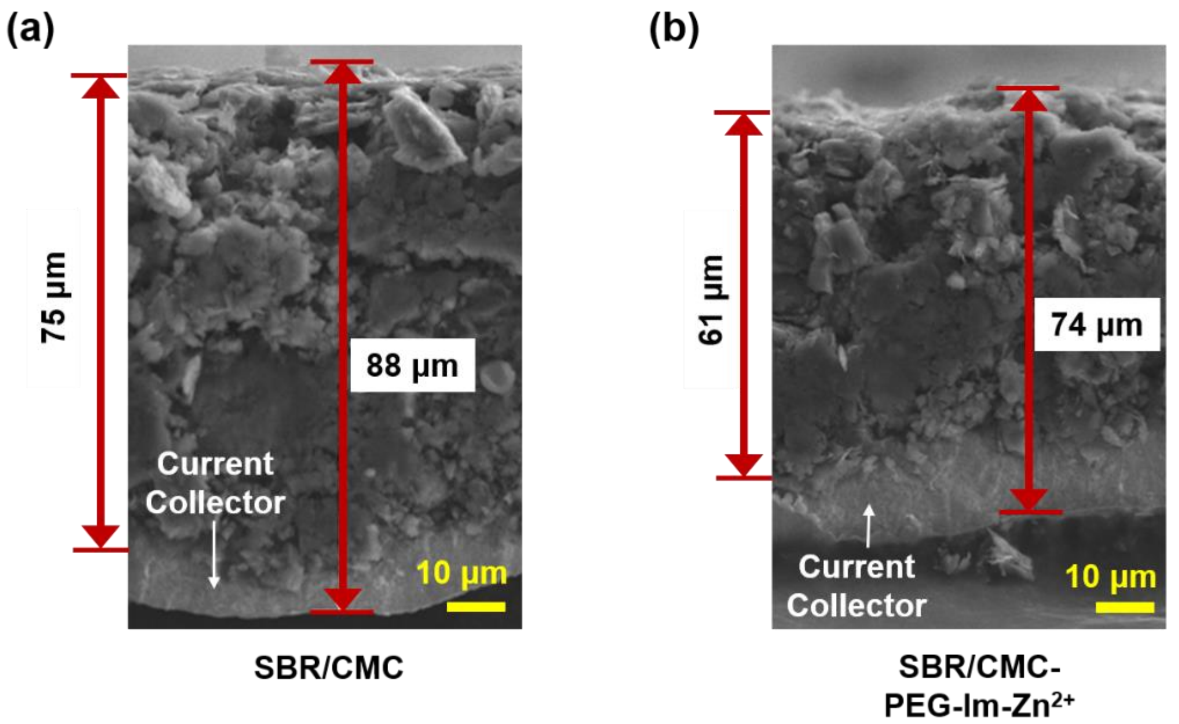

Figure S22. Cross-sectional SEM images of the (a) Si/C-SBR/CMC and (b) Si/C-SBR/**CMC-PEG-Im-Zn^{2+}** electrodes after the 135th delithiation.

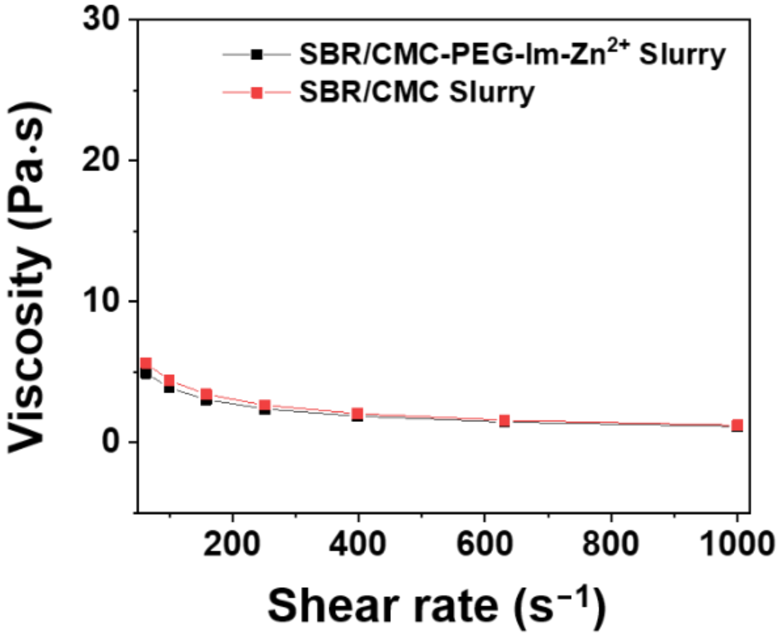

Figure S23. Viscosity *vs.* shear rate profiles for the SBR/**CMC-PEG-Im-Zn^{2+}** and SBR/CMC slurries.
